# Effects of the anesthesiologist’s experience on postoperative hoarseness after double-lumen endotracheal tube intubation: a single-center propensity score-matched analysis

**DOI:** 10.1186/s12871-020-01198-1

**Published:** 2020-11-05

**Authors:** Yuji Kamimura, Toshiyuki Nakanishi, Aiji Boku Sato, Satoshi Osaga, Eisuke Kako, Kazuya Sobue

**Affiliations:** 1 Department of Anesthesiology and Intensive Care Medicine, Nagoya City University Graduate School of Medical Sciences, 1 Kawasumi, Mizuho-cho, Mizuho-ku, Nagoya, 467-8601 Japan; 2 Department of Anesthesiology, Aichi Gakuin University School of Dentistry, 2-11 Suemori-dori, Chikusa-ku, Nagoya, 464-8651 Japan; 3 Clinical Research Management Center, Nagoya City University Hospital, 1 Kawasumi, Mizuho-cho, Mizuho-ku, Nagoya, 467-8601 Japan

**Keywords:** Tracheal intubation, Double-lumen endotracheal tube, Throat complication, Hoarseness, Trainee

## Abstract

**Background:**

Postoperative hoarseness after general anesthesia is associated with patient discomfort and dissatisfaction. A recent large retrospective study showed that single-lumen endotracheal tube intubation by a trainee did not alter the incidence of postoperative pharyngeal symptoms compared with intubation by a senior anesthesiologist. However, there is limited information about the relationship between the anesthesiologist’s experience and hoarseness after double-lumen endotracheal tube intubation. We tested the hypothesis that double-lumen endotracheal tube intubation performed by a trainee increases the incidence of postoperative hoarseness compared to intubation by a senior anesthesiologist.

**Methods:**

This retrospective observational study included patients who underwent lung resection between April 2015 and March 2018 at a university hospital. Double-lumen endotracheal tube intubation was carried out with a Macintosh laryngoscope. We divided the patients into 2 groups - one group comprised of patients who were intubated by a trainee anesthesiologist with < 2 years of experience, and the other group comprised of those who underwent intubation by a senior anesthesiologist with ≥2 years of experience. The primary outcome was the incidence of postoperative hoarseness 24 h after surgery and we collected data on postoperative hoarseness using a checklist of postanesthetic adverse events. One-to-one propensity score matching was conducted and *P* values < 0.05 were considered statistically significant.

**Results:**

There was a total of 256 eligible patients, of which 153 underwent intubation by trainee anesthesiologists, and the remaining 103 patients were intubated by a senior anesthesiologist. The one-to-one propensity score matching resulted in 96 pairs of patients for the groups. The incidence of postoperative hoarseness 24 h after surgery was significantly higher in patients who were intubated by a trainee anesthesiologist than in patients who were intubated by a senior anesthesiologist (9.4% vs. 2.1%, respectively; *P* = 0.03).

**Conclusions:**

Double-lumen endotracheal tube intubation by trainee anesthesiologists with < 2 years of experience increased the incidence of postoperative hoarseness 24 h after surgery compared to intubation by senior anesthesiologists with ≥2 years of experience.

**Supplementary Information:**

The online version contains supplementary material available at 10.1186/s12871-020-01198-1.

## Background

There is a correlation between postoperative hoarseness after general anesthesia and patient discomfort and dissatisfaction. Several risk factors, such as patient demographic factors, quality of intubation, and perioperative management, are reportedly associated with postoperative hoarseness [1–3].

Double-lumen endotracheal tube (DLT) intubation had been the gold-standard for surgical lung separation. However, the use of bronchial blockers is also an effective method for lung separation and has a lower incidence of postoperative hoarseness. This has led to an on-going debate regarding the best device for lung separation. A systematic review evaluating 307 patients from 4 studies showed that the use of DLTs was related to a higher risk of postoperative hoarseness than the use of a combination of single-lumen endotracheal tubes (SLTs) and endobronchial blockers [4]. The reported incidence of postoperative hoarseness after insertion of a DLT is 5 to 50% [4–6]. A high frequency of hoarseness may be caused by the thickness of the DLTs and the skills required for intubation.

The results of a recent large retrospective study including over 20,000 patients suggested that endotracheal intubation by a trainee did not increase postoperative throat symptoms compared to intubation by a senior anesthesiologist [7]. However, the study only included patients who underwent SLT intubation. Therefore, there is limited knowledge of the relationship between the anesthesiologist’s experience and hoarseness after DLT intubation.

In this study, we tested the hypothesis that DLT intubation by a trainee increases the incidence of postoperative hoarseness compared to DLT intubation by a senior anesthesiologist.

## Methods

The protocol for this study was approved by the Nagoya City University Graduate School of Medical Sciences and Nagoya City University Hospital Institutional Review Boards (Nagoya, Japan, approval number: 60-18-0073). According to our institutional review board’s code of ethics, we used an opt-out method and posted a description of the research protocol on the website of the Nagoya City University Graduate School of Medical Sciences on July 30, 2018, and the patients could withdraw from the study.

### Data source and study population

The present retrospective observational study included patients who underwent lung resection between April of 2015 and March of 2018. We included patients who underwent DLT intubation with a Macintosh laryngoscope and a neuromuscular blocking drug, who were ≥ 15 years of age, and who had an American Society of Anesthesiologists physical status classification (ASA-PS) of 1 or 2. Patients with preoperative hoarseness, those who were intubated with a video laryngoscope, those who required emergency surgery, and those with missing data were excluded from this study.

### Study variables

The exposure of interest was DLT intubation performed by a trainee or senior anesthesiologist. We divided patients into 2 groups: one group comprising patients who were intubated by a trainee anesthesiologist and the other comprising those who were intubated by a senior anesthesiologist. Anesthesiologists in Japan can only be certified as Qualified Anesthesiologists according to the Japanese Society of Anesthesiologists after completing a 2-year training program. Therefore, we defined trainee anesthesiologists as “anesthesiologists with less than 2 years of anesthesia experience” and senior anesthesiologists as “those with more than 2 years of anesthesia experience”. These definitions were the same as those used in a previous study [7]. We collected the following clinical variables: age, gender, height, weight, body mass index (BMI), ASA-PS, duration of anesthesia, intraoperative fluid balance, DLT size, intubation depth, number of intubation attempts, intracuff pressure of the DLT, Mallampati score, and Cormack–Lehane grade.

### Outcome measures

The primary outcome was incidence of postoperative hoarseness 24 h after surgery. Anesthesiologists in charge of postanesthetic rounds at our hospital must use a checklist of postanesthetic adverse events and determine the presence of hoarseness 24 h after surgery. The investigator (YK), who did not perform DLT intubation or manage anesthesia, collected data on postoperative hoarseness from electronic medical records using a checklist of postanesthetic adverse events. We defined postoperative hoarseness as “a patient-assessed change in voice quality”. We did not qualitatively or objectively evaluate postoperative hoarseness. We investigated whether the anesthesiologist who assessed postoperative hoarseness was the same one who provided anesthesia for the patient and whether he or she was a trainee or senior anesthesiologist.

### Perioperative patient treatment

There are no standardized methods for induction or maintenance of anesthesia. Electrocardiography, pulse oximetry, and invasive blood pressure monitoring were performed after patients arrived at the operating room. Patients received a combination of general and epidural anesthesia. General anesthesia was induced with propofol (a bolus dose of 1–2 mg/kg or a target-controlled infusion at 3–3.5 μg/ml), fentanyl (1–4 μg/kg) and remifentanil (0–0.3 μg/kg/min) following placement of a thoracic epidural catheter. An attending trainee or senior anesthesiologist performed DLT intubation with a Macintosh laryngoscope after bolus administration of rocuronium (0.6–1 mg/kg). Neuromuscular monitoring was not performed during tracheal intubation. Blade size (3 or 4) was chosen based on anesthesiologist preference and the patient’s physique. Portex® Blue Line® Endobronchial Tubes-left (Smiths Medical, Minneapolis, MN, USA) with a stylet were used in all procedures and a water-soluble lubricant without lidocaine was applied to the tube. We used a 37-Fr DLT for men and a 35-Fr DLT for women, but tube size was determined by the attending anesthesiologist based on the patient’s height [8]. The attending anesthesiologist guided the DLT into position via a flexible bronchoscope and assessed tube placement after changing patient to the lateral decubitus position. Anesthesia was maintained with 1–2.5% sevoflurane or propofol (target-controlled infusion at 2–3.5 μg/mL) and the Bispectral Index® value was kept between 40 and 60 throughout the entire procedure. Residual neuromuscular blockade was reversed with sugammadex (2–4 mg/kg), postoperatively, and the DLT was removed in the operating room.

### Statistical analysis

For sample size calculation, we assumed that the incidence of postoperative hoarseness 24 h after surgery in patients who underwent intubation by a trainee or senior anesthesiologist would be 20 and 5%, respectively, based on previous reports [4–6]. Thus, 89 patients in each group were required to provide 80% power to detect a statistical difference between groups using Fisher’s exact test with a two-sided significance level of 5%.

We conducted propensity score analyses to account for differences in baseline characteristics between the 2 groups. The c-statistic for evaluating goodness of fit was calculated and we performed one-to-one propensity score matching by nearest neighbor matching without replacement. Caliper width was set to 25% of the standard deviation of the propensity scores. Furthermore, the confounding factors used in the propensity score model were age, gender, height, weight, BMI, ASA-PS, duration of anesthesia, intraoperative fluid balance, tube size, tube depth, number of intubation attempts, intracuff pressure, Mallampati score, and Cormack–Lehane grade. We assessed the differences between the 2 groups before and after propensity score matching with standardized differences. Standardized differences of < 10% were considered negligible imbalances in the baseline characteristics between the 2 groups. We compared the incidence of hoarseness 24 h after surgery between the 2 groups using Fisher’s exact test for before matching and the McNemar test for after matching. A *P* value < 0.05 was considered statistically significant. All statistical analyses were performed using the R software package (version 3.5.0, R Foundation for Statistical Computing, Vienna, Austria).

## Results

**Fig. 1:**
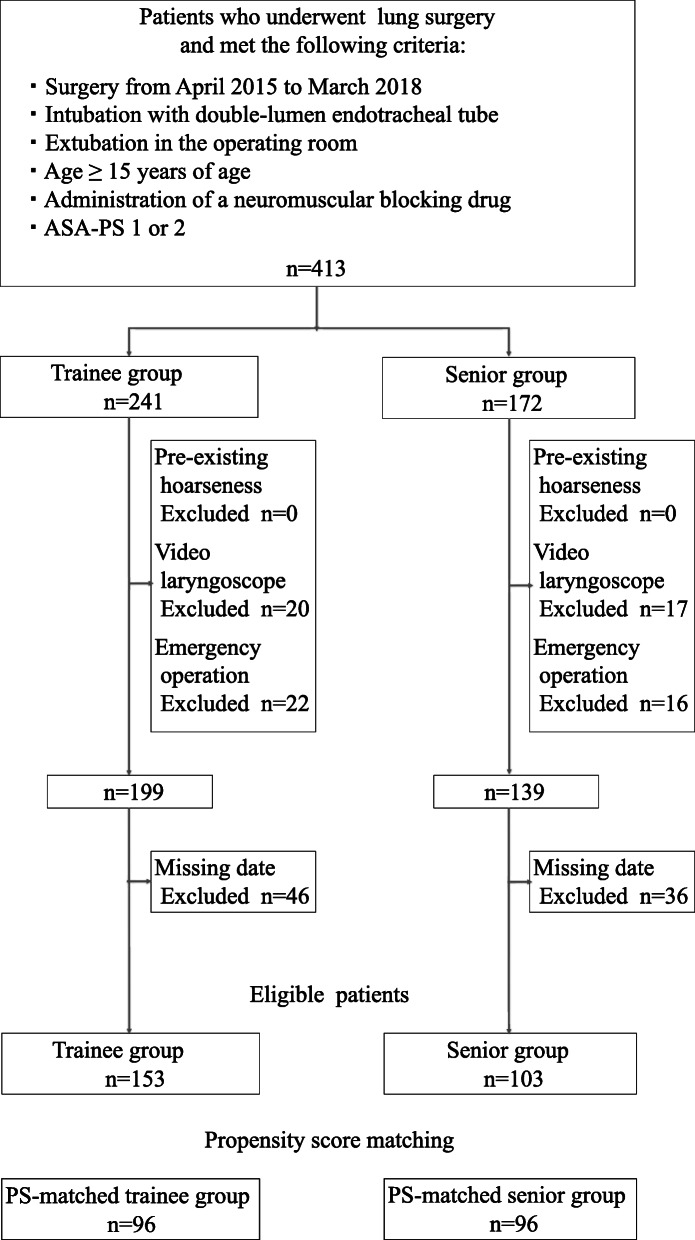
Study flow diagram. The values indicate the number of all eligible patients during the study period. ASA-PS, American Society of Anesthesiologists physical status classification; PS, propensity score

Figure 1 shows a flow diagram for cohort identification. We identified 413 lung cancer patients who underwent lung resection during the study period. Out of these patients, 256 were included in the full study cohort based on predetermined inclusion and exclusion criteria. These 256 patients included 153 patients who were intubated by a trainee anesthesiologist and 103 patients who were intubated by a senior anesthesiologist. Overall, 32 anesthesiologists (10 trainee anesthesiologists (listed in Supplementary Table S1) and 22 senior anesthesiologists) participated in this study. Median (interquartile range) length of experience was 1 year (1–2 years) for trainee anesthesiologists and 10 years (7–14 years) for senior anesthesiologists.

Table 1 shows patient characteristics prior to propensity score matching between the 2 groups. There was no significant difference between the 2 groups regarding the number of intubation attempts. Some characteristics, including age, weight, BMI, ASA-PS, intraoperative fluid balance, tube size, tube depth, intracuff pressure, Mallampati score, and Cormack–Lehane grade, had standardized differences of > 10%.

**Table 1:** Clinical characteristics prior to propensity score matching

	Prior to propensity score matching		
	Trainee	Senior	Standardized difference (%)
	*n* = 153	*n* = 103	
Age (median [IQR]; years)	69 [57, 75]	68 [61, 75]	15.5
Gender (male/female) (%)	90/63 (58.8/41.2)	62/41 (60.2/39.8)	2.8
Height (median [IQR]; cm)	161.4 [155.0, 168.4]	162.2 [155.6, 168.0]	0.6
Weight (median [IQR]; kg)	59.6 [52.6, 66.3]	58.0 [50.7, 64.3]	15
BMI (median [IQR]; kg/m^2^)	22.8 [20.5, 25.0]	22.3 [20.1, 24.2]	17.1
ASA-PS (%)			23.3
1	27 (17.6)	10 (9.7)	
2	126 (82.4)	93 (90.3)	
Duration of anesthesia (median [IQR]; h)	4.0 [3.1, 5.0]	4.0 [2.9, 5.0]	1.4
Intraoperative fluid balance (median [IQR]; ml)	1299 [995, 1733]	1232 [926, 1630]	14.8
Tube size (%)			14.6
32 Fr	12 (7.8)	6 (5.8)	
35 Fr	64 (41.8)	45 (43.7)	
37 Fr	69 (45.1)	49 (47.6)	
39 Fr	8 (5.2)	3 (2.9)	
Tube depth (median [IQR]; cm)	28 [27, 30]	29 [27, 30]	15.8
Intubation attempts (%)			4.1
1	147 (96.1)	99 (96.1)	
2	5 (3.3)	3 (2.9)	
3	0 (0.0)	0 (0.0)	
4	1 (0.6)	1 (1.0)	
Cuff pressure (median [IQR]; cmH_2_O)	20 [20, 20]	20 [20, 20]	10.4
Mallampati score (%)			17.1
1	109 (71.2)	81 (78.6)	
2	44 (28.8)	22 (21.4)	
3	0 (0.0)	0 (0.0)	
4	0 (0.0)	0 (0.0)	
Cormack–Lehane grade (%)			16.3
1	120 (78.4)	82 (79.6)	
2	31 (20.3)	21 (20.4)	
3	2 (1.3)	0 (0.0)	
4	0 (0.0)	0 (0.0)	

Data are described as frequency (%) or median [interquartile range, IQR]

*BMI* Body mass index; *ASA-PS* American Society of Anesthesiologists physical status classification

Table 2 shows patient characteristics after propensity score matching between the 2 groups. The established model for estimating propensity scores had a c-statistic of 0.635. A total of 96 patients from each group were matched through propensity score matching. Patient characteristics were well balanced between the 2 groups after matching and.

**Table 2:** Clinical characteristics after propensity score matching

	After propensity score matching		
	Trainee	Senior	Standardized difference (%)
	*n* = 96	*n* = 96	
Age (median [IQR]; years)	71 [63, 76]	68 [61, 75]	1.8
Gender (male/female) (%)	57/39 (59.4/40.6)	56/40 (58.3/41.7)	2.1
Height (median [IQR]; cm)	161.3 [154.6, 166.7]	162.1 [154.7, 167.9]	0.4
Weight (median [IQR]; kg)	57.5 [52.1, 61.97]	58.3[50.5, 64.2]	5.8
BMI (median [IQR]; kg/m^2^)	22.1 [20.1, 24.1]	22.4 [20.1, 24.3]	7.1
ASA-PS (%)			3.3
1	11 (11.5)	10 (10.4)	
2	85 (88.5)	86 (89.6)	
Duration of anesthesia (median [IQR]; h)	3.88 [2.75, 4.86]	3.95 [2.96, 5.04]	1.9
Intraoperative fluid balance (median [IQR]; ml)	1222 [931, 1518]	1224 [926, 1630]	3.1
Tube size (%)			7.8
32 Fr	5 (5.2)	6 (6.2)	
35 Fr	43 (44.8)	43 (44.8)	
37 Fr	45 (46.9)	45 (46.9)	
39 Fr	3 (3.1)	2 (2.1)	
Tube depth (median [IQR]; cm)	28 [27, 30]	28 [27, 30]	4.6
Intubation attempts (%)			14.5
1	93 (96.9)	92 (95.8)	
2	3 (3.1)	3 (3.1)	
3	0 (0.0)	0 (0.0)	
4	0 (0.0)	1 (1.0)	
Cuff pressure (median [IQR]; cmH_2_O)	20 [20, 22]	20 [20, 20]	6.5
Mallampati score (%)			2.5
1	74 (77.1)	75 (78.1)	
2	22 (22.9)	21 (21.9)	
3	0 (0.0)	0 (0.0)	
4	0 (0.0)	0 (0.0)	
Cormack–Lehane grade (%)			< 0.1
1	76 (79.2)	76 (79.2)	
2	20 (20.8)	20 (20.8)	
3	0 (0.0)	0 (0.0)	
4	0 (0.0)	0 (0.0)	

Data are described as frequency (%) or median [interquartile range, IQR]

*BMI* Body mass index; *ASA-PS* American Society of Anesthesiologists physical status classification

the incidence of postoperative hoarseness 24 h after surgery was significantly higher for intubation by a trainee anesthesiologist than for intubation by a senior anesthesiologist (9.4% vs. 2.1%, *P* = 0.03; Table 3). There were no patients with surgical recurrent nerve injury or continuous hoarseness who required referral to an otolaryngologist in either group. Postoperative hoarseness was determined by the anesthesia provider in 85% of trainee intubations and 80% of senior anesthesiologist intubations. There were no patients who could not be evaluated because they had a Glasgow Coma Scale < 15 or Numerical Rating Scale > 5.

**Table 3:** Incidence of postoperative hoarseness 24 h after surgery

	Full cohort			Propensity score-matched cohort		
Outcome, n (%)	Trainee	Senior	*P*	Trainee	Senior	*P*
	*n* = 153	*n* = 103		*n* = 96	*n* = 96	
Hoarseness	18 (11.8)	2 (1.9)	0.004	9 (9.4)	2 (2.1)	0.03

Data are described as frequency (%)

We also compared the incidence of postoperative hoarseness between the first 1–5 cases and after the sixth and subsequent cases for each trainee anesthesiologist. There was no significant difference between the 2 groups (Supplementary Table S1). We also compared the incidence of postoperative hoarseness between Cormack–Lehane grade 1 and 2, but found no significant difference (Supplementary Table S2).

## Discussion

Patients who underwent DLT intubation by a trainee anesthesiologist with < 2 years of experience had a higher incidence of postoperative hoarseness than those who underwent DLT intubation by a senior anesthesiologist with ≥2 years of experience in lung surgery. This result suggests that lack of experience could be a risk factor for postoperative hoarseness in patients undergoing DLT intubation.

The increased incidence of postoperative hoarseness observed in our patients who were intubated by a trainee anesthesiologist differed from the results of a previous study using SLTs [7]. One possible explanation for this difference may be that DLT intubation requires more technical skills than SLT intubation for the following reasons. First, the thicker diameter of DLTs may have made it difficult for trainee anesthesiologists to pass them through the glottis. The incidence of postoperative hoarseness was reported to directly correlate with endotracheal tube size [3]. Second, a DLT has a solid curved body, which can easily come into contact with the vocal cords [9]. During DLT intubation by a trainee anesthesiologist, therefore, the tube may more easily and frequently come into contact with the vocal cords than in intubations by senior anesthesiologists. There was no difference in the number of intubation attempts between trainee and senior anesthesiologists, but there might have been more strain on the vocal cords when trainee anesthesiologists used DLTs. The difference in the incidence of postoperative hoarseness between trainee and senior anesthesiologists, despite adjustments for the number of intubation attempts and tube size, suggests that an unseen skill level may account for the incidence of postoperative hoarseness.

The incidence of postoperative hoarseness 24 h after surgery was lower in both groups in the present study (9.4% for trainee anesthesiologists and 2.1% for senior anesthesiologists) than that in previous studies (5 to 50%) [4–6]. Only patients who subjectively complained were considered to have postoperative hoarseness, and therefore the incidence of postoperative hoarseness may have been underestimated. Thus, it is not easy to compare the results of this study to those of previous studies because of the different definitions of hoarseness. It is essential to know patient comfort level bcause postoperative hoarseness is a subjective patient complaint. Therefore, we believe that the outcome assessed in our study is clinically meaningful. A validated outcome measure, such as voice handicap index [10], may be a more reliable assessment in future studies.

Secondary analyses showed that the first 1–5 intubations for each trainee anesthesiologist, and Cormack–Lehane grade, were not associated with a significant increased risk of postoperative hoarseness 24 h after surgery in patients who underwent DLT intubation. However, postoperative hoarseness tended to be higher in the first 1–5 cases and in Cormack–Lehane grade 2 patients. Since the relatively small sample size of our study cannot provide adequate power for these comparisons, further study is needed to confirm these results.

We acknowledge that this study had some limitations. First, it was a single-center, retrospective observational study with relatively small sample size. Prospective randomized controlled trials are required to validate our results in the future. Second, a significant number of patients were excluded from this study, which may have led to selection bias. Third, we defined trainee anesthesiologists as “anesthesiologists with less than 2 years of anesthesia experience” and senior anesthesiologists as “those with more than 2 years of anesthesia experience”. It may be difficult to apply our results directly to other countries even though these definitions were equivalent to those used in a previous study [7]. Fourth, neuromuscular monitoring was not performed during tracheal intubation. The difference between trainee and senior anesthesiologists regarding the depth of muscle relaxation might have affected the incidence of hoarseness. Fifth, 80–85% of the evaluators were anesthesia providers, who were not blinded and may have caused observer bias and ascertainment bias. Moreover, it cannot be ruled out that trainee anesthesiologists may have more aggressively assessed the patient’s hoarseness. However, this study has the advantage that neither the evaluators nor the patients were aware of the study’s purpose due to the study’s retrospective nature. Therefore, evaluator influence on the results of this study, which were analyzed in real-world clinical practice, is likely minimal. Finally, although we attempted to limit selection bias using propensity score matching, the multifactorial etiologies of postoperative hoarseness that affect the outcomes may not have been removed.

## Conclusions

DLT intubation by trainee anesthesiologists with < 2 years of experience increased the incidence of postoperative hoarseness 24 h after surgery compared with DLT intubation by senior anesthesiologists with ≥2 years of experience.

## Supplementary Information

**Additional file 1:** **Table S1.** Details of intubations performed by trainees and incidence of postoperative hoarseness. **Table S2.** Incidence of postoperative hoarseness in patients with Cormack–Lehane grade 1 and 2.

## Data Availability

The datasets used and/or analyzed during the current study are available from the corresponding author upon reasonable request.

## Abbreviations

ASA-PS: American Society of Anesthesiologists physical status classification
BMI: Body mass index
DLT: Double-lumen endotracheal tube
SLT: Single-lumen endotracheal tube
