# Supplementary material for: Effects of the anesthesiologist’s experience on postoperative hoarseness after double-lumen endotracheal tube intubation: a single-center propensity score-matched analysis
Source: BMC Anesthesiol. 2020 Nov 5;20:278. doi: 10.1186/s12871-020-01198-1 (PMC7643342; doi:10.1186/s12871-020-01198-1)
Supplement: Supplementary file 1 — Additional file 1: Table S1. Details of intubations performed by trainees and incidence of postoperative hoarseness. Table S2. Incidence of postoperative hoarseness in patients with Cormack–Lehane grade 1 and 2. [file 12871_2020_1198_MOESM1_ESM.docx]

**Supplementary Tables**

Supplementary Table S1. Details of intubations performed by trainees and incidence of postoperative hoarseness.

| Trainee | Number of cases | Hoarseness in 1–5 cases | Hoarseness after the 6 cases | *P* |
| --- | --- | --- | --- | --- |
| A | 13 | 1/5 | 0/8 |  |
| B | 18 | 2/5 | 1/13 |  |
| C | 16 | 0/5 | 1/11 |  |
| D | 15 | 0/5 | 0/10 |  |
| E | 10 | 2/5 | 1/5 |  |
| F | 25 | 2/5 | 0/20 |  |
| G | 23 | 0/5 | 4/18 |  |
| H | 14 | 1/5 | 1/9 |  |
| I | 9 | 0/5 | 1/4 |  |
| J | 10 | 0/5 | 1/5 |  |
| Total | 153 | 8/50(16.0%) | 10/103(9.7%) | 0.29 |

Data are described as frequency (%). Mantel–Haenszel test was used and a P value <0.05 was considered statistically significant.

Supplementary Table S2. Incidence of postoperative hoarseness in patients with Cormack–Lehane grade 1 and 2.

| Anesthesiologist’s experience | CL1 | CL2 | *P* |
| --- | --- | --- | --- |
| Trainee | 6/76 (7.8%) | 3/20 (15.0%) | 0.39 |
| Senior | 1/76 (1.3%) | 1/20 (5.0%) | 0.38 |
| Total | 7/152 (4.6%) | 4/40 (10.0%) | 0.19 |

Data are described as the frequency (%). Fisher’s exact test and Mantel–Haenszel test were used for subgroup analysis of trainee and senior anesthesiologists and stratified analysis, respectively. P values <0.05 were considered statistically significant.

CL1, Cormack–Lehane grade 1; CL2, Cormack–Lehane grade 2
